# Peripapillary capillary vessel density progression in advanced glaucoma: a case report

**DOI:** 10.1186/s12886-018-1021-x

**Published:** 2019-01-05

**Authors:** Gábor Holló

**Affiliations:** Department of Ophthalmology, Semmelweis University, Mária u. 39, Budapest, 1085 Hungary

**Keywords:** AngioVue optical coherence tomography angiography, Floor effect, Glaucoma, Peripapillary vessel density, Progression, Retinal nerve fiber layer thickness

## Abstract

**Background:**

We report a case of advanced juvenile open-angle glaucoma (JOAG) in which peripapillary capillary vessel density (PcVD) in the inferior retina showed significant progression while the spatially corresponding retinal nerve fiber layer thickness (RNFLT) and visual field cluster defect values had reached their minimal detectable values, and showed no change during the follow-up (floor effect).

**Case presentation:**

A 45-year old white female patient with very advanced under treatment JOAG in the left eye was prospectively investigated with the AngioVue OCT (Optovue Inc., Fremont, USA) for RNFLT and PcVD, and Octopus Normal G2 visual field testing, at 6-month intervals for 2.5 years (6 visits). Images quality was high (8/10 in 5 visits and 7/10 in one visit), and the optical media were clear. For the superior and inferior retina the baseline RNFLT and PcVD values were 48 and 43 μm, and 28.9 and 36.5%, respectively. Using the instrument’s linear regression analysis significant progression (*P* < 0.05) was seen only for the hemifield with greater baseline RNFLT (superior RNFLT: − 0.5 μm/year) and the hemifield with greater baseline PcVD (inferior PcVD: − 2.4%/year). All inferior visual field cluster defect values progressed significantly (2.0 to 5.1 dB/year) while in the superior clusters no progression was measurable due to software indicated floor effect.

**Conclusion:**

Our case shows that PcVD progression can be measured in advanced glaucoma, that PcVD can show floor effect, and that it may indicate glaucomatous progression when the spatially corresponding RNFLT and visual field cluster defect do not show progression due to floor effect.

## Background

In the last 4 years optical coherence tomography angiography (OCTA) has been intensively investigated for clinical usefulness in glaucoma [1, 2]. While the various OCTA instruments and techniques proved their capability to separate normal eyes from glaucoma eyes [1–6] little information became available on the role of OCTA in glaucoma follow-up and its ability to measure glaucomatous progression [7–13]. Regarding glaucomatous progression most OCTA results were published with different software versions of the Angiovue OCTA (Optovue Inc., Fremont, CA, USA). It was shown that reduced peripapillary angioflow vessel density is a risk factor of further retinal nerve fiber layer thickness (RNFLT) progression [9]. Using a recently released Angiovue OCTA software update (2017.1 software version with Phase 7 update) it became possible to selectively measure peripapillary capillary vessel density (PcVD) and its sector values [10]. PcVD is the perfused capillary area expressed as a percentage of the total examined area or its sectors, respectively, in the radial peripapillary capillaries layer, which corresponds to the retinal nerve fiber layer [10]. It does not contain information arriving from larger retinal vessels. Using all-vessel peripapillary angioflow measurements and selective PcVD measurements it was shown that detection of peripapillary vessel density progression is possible in glaucoma [8, 10–12], and that breath holding during image acquisition does not influence the PcVD measurement results [13]. The above data, however, were obtained in healthy, early and moderate glaucoma eyes. The applicability of PcVD for the detection and measurement of progression in very late stages of glaucoma, when RNFLT and routine visual field testing no longer provide useful information on the deterioration in the patient’s condition due to floor effect [14], has not yet been evaluated.

In the current case report we present the result of a 2.5-year prospective RNFLT, PcVD, and Octopus perimeter visual field cluster progression analysis on an advanced juvenile open-angle glaucoma eye for which the measured inferior RNFLT and the spatially corresponding superior visual field cluster values did not alter due to floor-effect, while inferior PcVD showed significant progression.

## Case presentation

In 2008 a 35-year old female patient was referred to the Glaucoma Center of the Semmelweis University in Budapest, where advanced juvenile open-angle glaucoma was diagnosed in both eyes. Her untreated intraocular pressure (IOP) was 36 and 28 mmHg, visual acuity eccentric hand motion and 1.0, and central corneal thickness 531 and 542 μm on the right and left eye, respectively. The vertical cup/disc ratio was 0.95 in both eyes. A fixed combination of bimatoprost and timolol was prescribed, and the under treatment IOP became controlled for both eyes. Over the next 10 years the under treatment IOP of the left eye ranged between 9 and 14 mmHg (typically 12 to 13 mmHg). The patient entered a long-term, prospective glaucoma structure-function investigation in the Glaucoma Center of the Semmelweis University in Budapest, for which the research protocol was approved by the Institutional Review Board for Human Research of Semmelweis University, Budapest and written informed consent was given by the patient before enrolment. The left eye was followed with various imaging methods and the Octopus 30-degree normal G2 visual field test (Octopus 900 perimeter, Haag-Streit AG, Koeniz-Berne, Switzerland) at regular 6-month intervals. Peripapillary OCTA measurement with the Angiovue OCT via undilated pupil became a part of the tests in March 2015, and was performed in all study visits at 6-month intervals until December 2017 (2.5-year follow-up and 6 visits). The peripapillary imaging was made with software version 2015.100.0.33, and it was analyzed with the 2017.1 software version and the Phase 7 update [10]. The 10-cluster progression analysis function of the Octopus perimeter was used to match functional progression to structural progression [15, 16]. All visual field tests had less than 20% false positive and less than 20% false negative response rates.

**Fig. 1:**
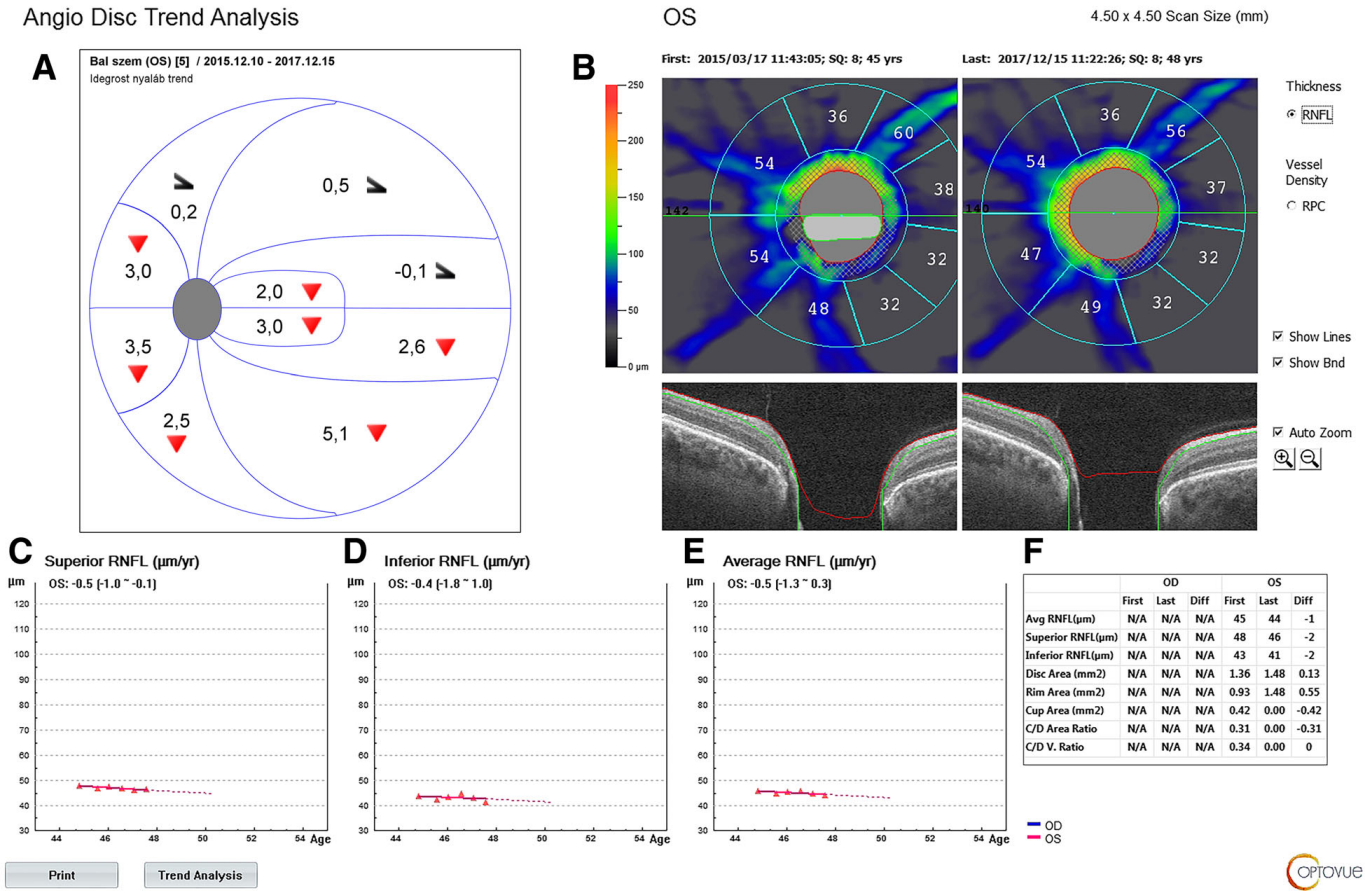
The Angiovue OCT progression report for inferior and superior retinal nerve fiber layer (RNFL) thickness, and the corresponding Octopus visual field cluster progression analysis report for the current case (2.5-year follow-up). **a**. Octopus visual field cluster progression analysis report: the red triangles indicate significant cluster mean defect progression for which the values are given in each cluster in dB/year, respectively. The black half-arrowhead symbol indicates floor effect for the corresponding clusters. **b**. first and last peripapillary RNFL images; **c**. superior peripapillary RNFL thickness progression; **d**. inferior peripapillary RNFL thickness progression; **e**. average peripapillary RNFL thickness progression; **f**, thickness parameter values measured during the first and last visits, and their differences

**Fig. 2:**
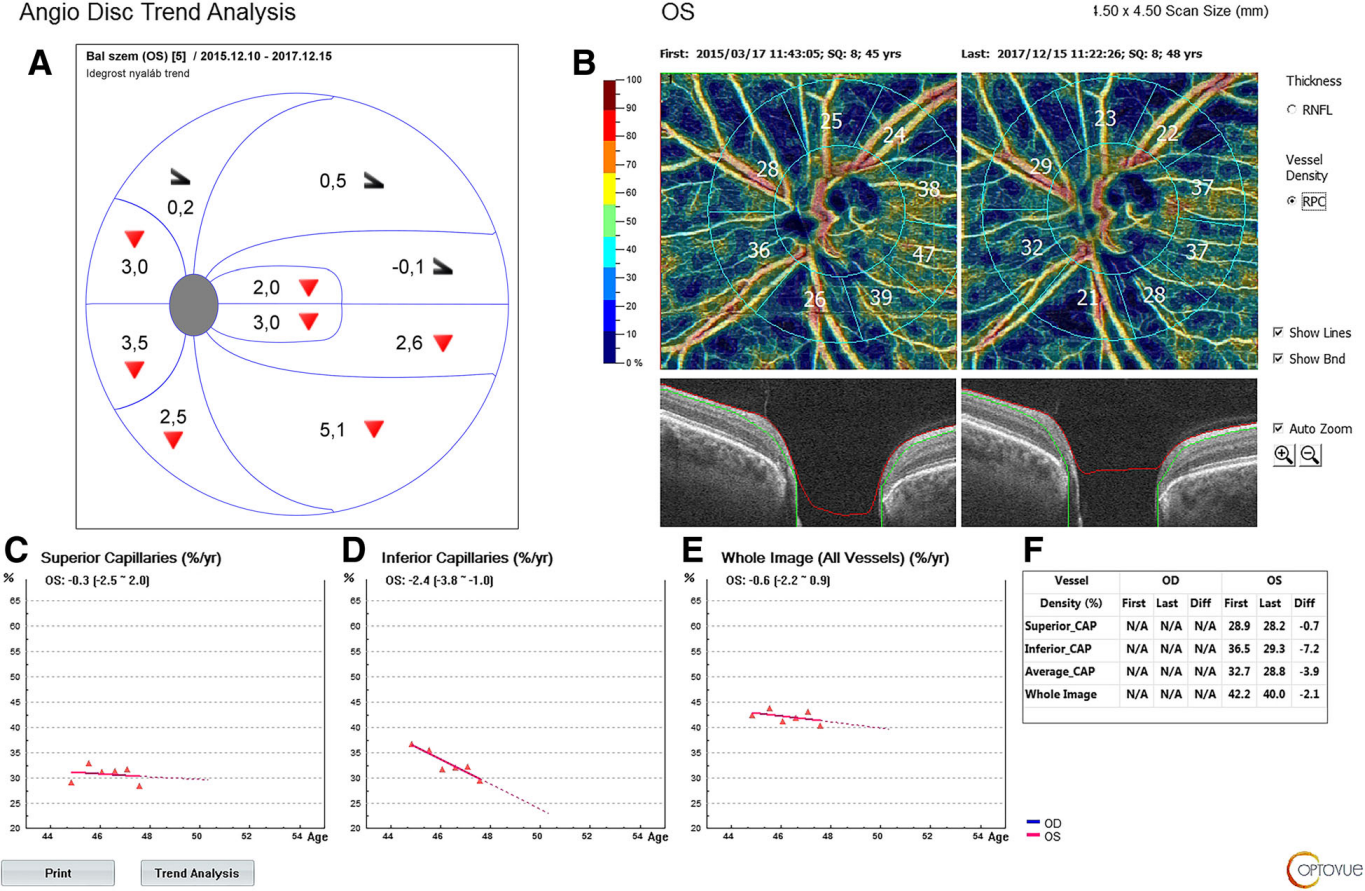
The Angiovue OCT progression report for inferior and superior peripapillary capillary vessel density (PcVD), and the corresponding Octopus visual field cluster progression analysis report for the current case (2.5-year follow-up). **a** Octopus visual field cluster progression analysis report: the red triangles indicate significant cluster mean defect progression for which the values are given in each cluster in dB/year, respectively. The black half-arrowhead symbol indicates floor effect for the corresponding clusters. **b** first and last peripapillary vessel density images; **c**. superior PcVD progression; **d**. inferior PcVD progression; **e**. whole image all-vessel density progression; **f**. vessel density parameter values measured during the first and last visits, and their differences

For PcVD and RNFLT progression analysis only high quality images with no artifacts or vitreous floaters were used. The image quality score was 8/10 for all but one image, for which the score was 7/10. All image acquisitions were made by the same investigator (GH). For PcVD measurements split-spectrum amplitude-decorrelation angiography was used. Motion correction was applied and the eye tracking function was activated. The 4.5 mm × 4.5 mm scan size was used. The peripapillary area was automatically defined as the area between the 2 and 4 mm diameter elliptical contour lines automatically fitted around the disc margin [10]. RNFLT was automatically determined as a part of peripapillary OCTA measurement. For progression analysis both RNFLT and PcVD are graphically presented and statistically evaluated with linear regression analysis, for the inferior and superior 180-degree retinal areas, respectively (Figs. 1 and 2). No exact *P*-value is given, significant progression is defined as *P* < 0.05.The software version also provides information on 360-degree PcVD, and total image area all-vessels density.

At the beginning of the OCTA follow-up the visual field mean defect was 17.1 dB. The superior and inferior RNFLT values were 48 and 43 μm (Fig. 1), and the corresponding PcVD values 28.9 and 36.5% (Fig. 2), respectively. During the follow-up period the uncorrected visual acuity remained unchanged (1.0). The rate of change was similar for the superior and inferior RNFLT, but only the superior RNFLT (which at the beginning of the follow-up was 5 μm thicker than the inferior RNFLT) progressed in a statistically significant manner (− 0.5 μm/year). In contrast, superior PcVD remained stable, but inferior PcVD (which was 7.6% higher than superior PcVD at the baseline visit) progressed significantly at a rate of − 2.4% per year. The difference between the first and last visits was − 0.7% for the superior and − 7.2% for the inferior PcVD (Fig. 2). The Octopus visual field cluster analysis showed that the inferior clusters all progressed significantly at a rate of 2.0 to 5.1 dB/year, which spatially corresponds with the superior RNFLT progression. But for the superior visual field clusters no progression was detectable due to floor effect, as indicated by the software with the black half-arrowhead symbols which appear in Figs. 1 and 2. This corresponds with the apparent stability of the very low inferior RNFLT, which is probably also caused by floor effect. No progression was detected either for the superior hemifield inner macular retinal thickness (ganglion cell complex, GCC; Pearson correlation, *P* = 0.638) or for the inferior hemifield GCC (*P* = 0.139).

## Discussion and conclusions

In the presented case we prospectively followed a very advanced juvenile open-angle glaucoma eye using peripapillary RNFLT, PcVD and visual field progression analysis for 2.5 years (6 visits with 6-month separation). Thanks to the patient’s relative youth, clear optical media and good cooperation the visual field data were reliable and all images were of high quality. Not unexpectedly we found statistically significant RNFLT and visual field cluster progression. But - also not unexpectedly - the progression was detectable only where the baseline parameter values made it technically possible to detect deterioration. RNFLT was very low for both hemifields, but progression was significant only for superior RNFLT, which was 5 μm higher at baseline. The spatially corresponding inferior visual field clusters progressed significantly, which supports the conclusion that the superior RNFLT progression was true progression. Indirectly, a similar confirmation was seen for the very low and apparently stable inferior RNFLT and the spatially corresponding superior visual field clusters for which progression was not measurable due to floor effect [14, 16].

The novelty of this case report lies in the progression measured for the hemifield PcVD values. First, similarly to the hemifield RNFLT values the hemifield PcVD values were very low, approximately 60% of the usual normal value [10]. Significant progression was found only for the inferior PcVD, which was 7.6% higher than the superior PcVD at baseline. This pattern suggests that a floor effect can be present for PcVD and in advanced glaucoma PcVD progression may be better detected in areas where the baseline value is higher. Second, our case shows that the floor effects for hemifield peripapillary RNFLT measurement and for hemifield PcVD measurement can be separated from each other. In the current case we measured statistically significant progression for the relatively preserved RNFLT in the superior hemifield where the very low baseline PcVD remained stable, and we found statistically significant progression for the relatively preserved PcVD in the inferior hemifield where the very low baseline RNFLT remained stable. Though certain macular parameters may indicate glaucomatous progression when RNFLT progression remains undetected due to advanced glaucoma [17], in the current case no time-dependent change was found for either GCC hemifield value.

In conclusion, our case report suggests that PcVD progression can be measurable in advanced glaucoma, that it can show floor effect in progression analysis, and that its floor effect may appear in retinal areas which are spatially different from those with RNFLT floor effect. To better understand PcVD progression in advanced open-angle glaucoma and to clarify the potential usefulness of PcVD progression analysis in the management of advanced glaucoma sufficiently powered prospective investigations are necessary.

## Abbreviations

IOP: intraocular pressure
JOAG: juvenile open-angle glaucoma
OCTA: optical coherence tomography angiography
PcVD: peripapillary capillary vessel density
RNFLT: retinal nerve fiber layer thickness
